# Adoptive Cell Transfer of Allogeneic Epstein–Barr Virus-Specific T Lymphocytes for Treatment of Refractory EBV-Associated Posttransplant Smooth Muscle Tumors: A Case Report

**DOI:** 10.3389/fimmu.2021.727814

**Published:** 2021-12-03

**Authors:** Bjoern-Thore Hansen, Petra Bacher, Britta Eiz-Vesper, Steffen M. Heckl, Wolfram Klapper, Karoline Koch, Britta Maecker-Kolhoff, Claudia D. Baldus, Lars Fransecky

**Affiliations:** ^1^ Medical Department II – Hematology and Oncology, University Hospital Schleswig-Holstein, Kiel, Germany; ^2^ Institute of Immunology, Christian-Albrechts-University of Kiel, Kiel, Germany; ^3^ Institute of Clinical Molecular Biology, Christian-Albrechts-University of Kiel, Kiel, Germany; ^4^ Institute of Transfusion Medicine and Transplant Engineering, Hannover Medical School, Hannover, Germany; ^5^ Section for Hematopathology and Lymph Node Registry, Department of Pathology, University Hospital Schleswig-Holstein, Kiel, Germany; ^6^ Department of Pediatric Hematology and Oncology, Hannover Medical School, Hannover, Germany

**Keywords:** posttransplant smooth muscle tumors, PTSMT, smooth muscle tumor, adoptive cell transfer, virus-specific T cells, alloCELL, T-cell transfer, case report

## Abstract

Posttransplant smooth muscle tumors (PTSMTs) are rare Epstein–Barr virus (EBV)-associated neoplasms, mostly occurring after solid organ transplantation. Current therapeutic strategies include surgery and reduction of immunosuppressive medication. We describe for the first time a novel treatment approach for PTSMT by adoptive cell transfer (ACT) of EBV-specific T cells to a 20-year-old patient with a medical history of cardiac transplantation, posttransplant lymphoproliferative disease, and multilocular PTSMT. During ACT, mild cytokine release syndrome occurred, while no unexpected safety signals were recorded. We observed *in vivo* expansion of EBV-specific T cells and reduction of EBV viremia. Best response was stable disease after 4 months with reduction of EBV viremia and normalization of lactate dehydrogenase levels. ACT with EBV-specific T cells may be a safe and efficacious therapeutic option for PTSMT that warrants further exploration.

## Introduction

Adoptive cell transfer (ACT) of virus-specific T cells (VSTs) from healthy donors has been successfully used for the treatment for transplantation-associated diseases, which were refractory to first-line therapy, including Epstein–Barr virus (EBV)-associated posttransplant lymphoproliferative disease (PTLD) (1–5) and cytomegalovirus (CMV) (4, 6) and human adenovirus (HAdV) (4, 7–9) infections. In addition, the use of ACT in the context of infection prophylaxis (2, 10, 11) or consolidation treatment (5) has been suggested.

ACT is associated with high levels of disease response (11) while generally being well-tolerated, reflected by a rate of reported adverse events of 4% (n = 475) (7, 11) that include local swelling of the tumor site (2), aggravation of preexisting graft-versus-host disease (GvHD) (9, 10), *de novo* development of GvHD (4, 8, 10, 12), and notable cytokine release syndrome (CRS) grades 2–3 (13).

EBV is a human herpesvirus that can drive the pathogenesis of both lymphoma and carcinoma. EBV can be detected in up to 30% of Hodgkin’s lymphomas, in 5%–10% of diffuse large B-cell lymphomas (DLBCLs), and in the vast majority of plasmablastic lymphomas of immunocompromised patients (14). Important viral proteins include Epstein–Barr nuclear antigen 1 (EBNA-1) and EBNA-2, which play important roles for intracellular persistence of the viral genome, acting as a transcription factor with crosstalk to PI3K/Akt/mTor (phosphatidylinositol 3-kinase/protein kinase B/mammalian target of rapamycin) signaling and for proliferation and survival of infected B cells. In addition, EBV is characterized by functional RNAs called EBV small encoded RNA molecules (EBER), whose precise function remains largely unknown (14).

EBV-associated smooth muscle tumors (SMTs) are rare neoplasms of indefinite malignancy linked to states of immunosuppression. Classification differentiates SMT after solid organ transplantation (PTSMT), SMT with the human immunodeficiency virus (HIV-SMT), and SMT with congenital immunodeficiency (CI-SMT). For patients with PTSMT, medical history of PTLD is not uncommon and most often associated with solid organ transplantation (15). It is assumed that PTSMT originates from EBV-infected smooth muscle cells (SMCs) of venous walls (16). While there is some evidence that the EBV receptor CD21, which is expressed by SMC, plays an important role in infection and transformation (17, 18), cases with CD21^-^ PTSMT cells have been described (19, 20). This implicates that there are different trajectories toward malignant degeneration in SMT (21).

Symptoms of PTSMT are mainly based on the location of the tumor(s), including pain and organ dysfunction. Diagnosis is most frequently made by sonography or computed tomography (CT) with subsequent needle or laparoscopic biopsy. In case of colonic involvement, colonoscopy reveals the classical phenotype of PTSMT.

Of 36 previously published cases, eight (22%) were monocentric SMTs, while 28 (78%) showed multilocular occurrence, which mostly evolved in a synchronous manner (19, 20, 22–31).

While therapeutic standards are currently lacking, the therapeutic mainstay consists of surgery, decreasing immunosuppression, and medical therapies. Surgery is the therapy of choice but is only achievable in about 25% of the cases (22, 25, 28–30). Tapering of the immunosuppressants is pivotal for disease control and should be attempted in all patients, especially since responses have been achieved by dose modification only in selected cases (2 of 36, 5.5%), indicating immunological antitumor effects like those seen with PTLD (20, 24).

In four of the 36 published cases, tapering of immunosuppression was combined with different chemotherapy regimens. Cytotoxic drugs included vincristine (19), dactinomycin (19), and cyclophosphamide (19), trabectedin (24), gemcitabine (24), temozolomide (23), and isotretinoin (26). Although a systematic analysis is lacking, reported effectiveness was very limited, with response rates below 10%.

Of note, the mTOR inhibitor sirolimus was successfully used to achieve complete remissions in four out of six PTSMT patients with a mean follow-up of 3.8 years (28, 30, 31). Here, sirolimus may interfere with the malignant transformation in SMT, as the PI3K/Akt/mTOR pathway plays a crucial role in SMTs (32).

Prognosis of PTSMTs was defined as a function of therapeutic regimen, cerebral involvement, onset time, and accompanying diseases (24). Median overall survival was estimated at 6 months when chemotherapy or radiotherapy was initiated, 28.5 months when reduction of immunosuppression sufficed, and up to 108 months when surgery and reduction of immunosuppression were realized (33).

**Figure 1:**
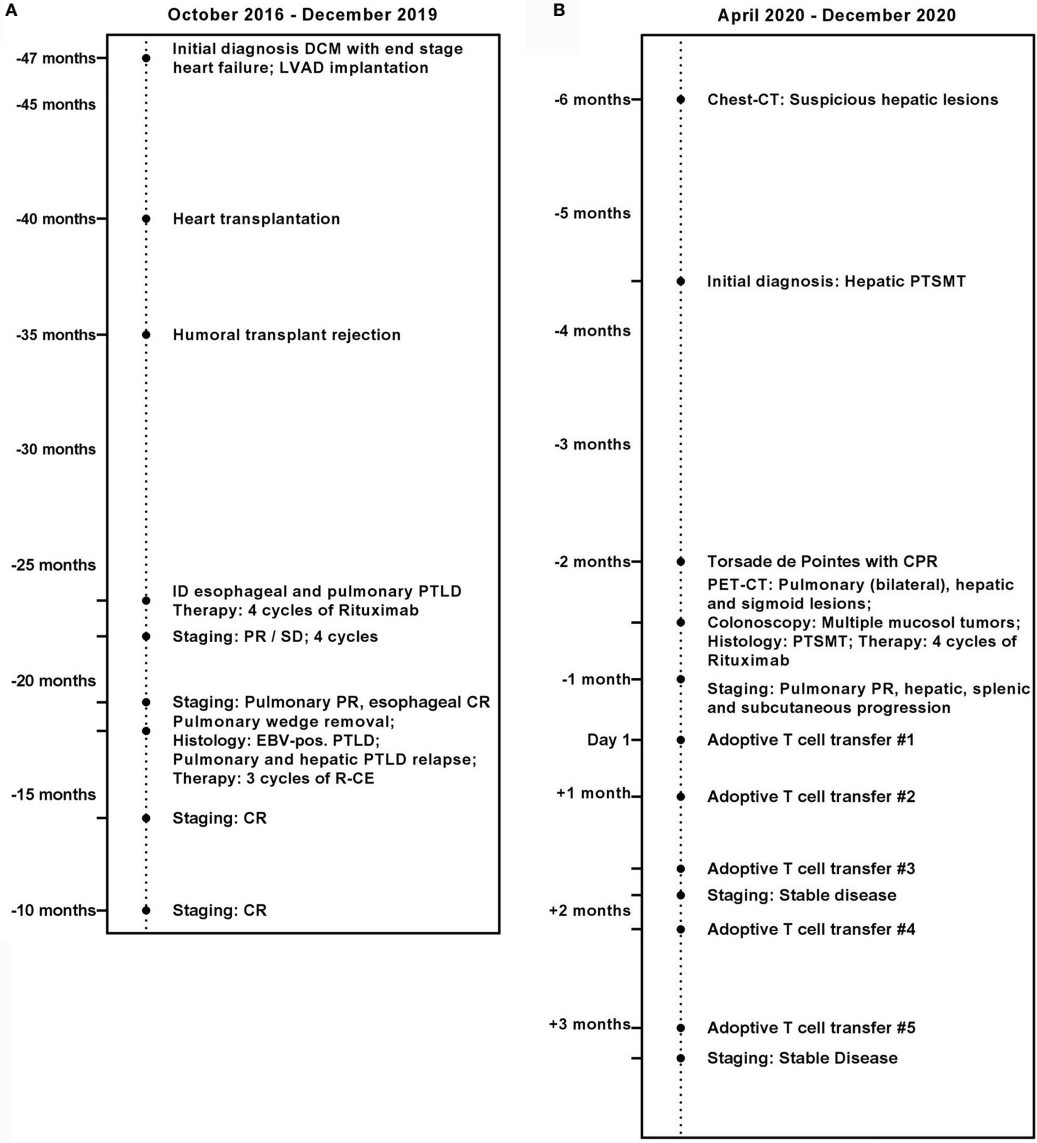
Medical history of the patient. **(A)** October 2016 until December 2019. **(B)** April 2020 until December 2020.

Here, we present a case of a 20-year-old female with refractory multilocular PTSMT (**Figure 1**). The patient underwent cardiac transplantation in 2017 after diagnosis of a dilated cardiomyopathy and borderline myocarditis with end-stage heart failure in 2016. After transplantation, she received prophylactic immunosuppression with everolimus and tacrolimus. In 2018, the patient was diagnosed with EBV^+^ CD19^+^ CD30^+^ polymorphic PTLD with pulmonary manifestations and monomorphic PTLD with the histological picture of EBV^+^ DLBCL in an esophageal biopsy. The patient received four courses of rituximab after tapering of the immunosuppression in October 2018. Due to poor response, therapy was augmented with CHOP (cylophosphamide, hydroxydaunorubicin, vincristine (oncovin), prednisolone) polychemotherapy without vincristine and three courses of rituximab, carboplatin, and etoposide, which resulted in complete remission with an event-free survival of 12 months.

**Figure 2:**
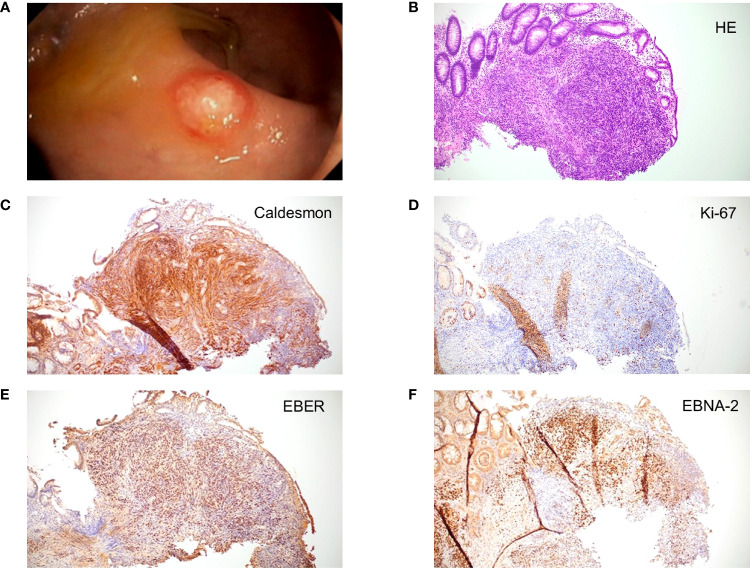
Macroscopic and microscopic images of posttransplant smooth muscle tumor (PTSMT). **(A)** Colonoscopic image of PTSMT depicted as a nodular mucosal tumor with erythematous margin and a mucous cap. **(B–F)** Histopathological stainings from the tumor depicted in panel **(A)**, ×100 magnification applied. Staining: **(B)** hematoxylin and eosin stain; **(C)** caldesmon stain; **(D)** Ki-67 stain; **(E)** Epstein–Barr virus-encoded small RNAs (EBER) *in situ* hybridization; **(F)** Epstein–Barr virus nuclear antigen 2 (EBNA-2) stain.

In April 2020, CT revealed new suspicious hepatic lesions. Needle biopsy of the liver revealed the diagnosis of EBV^+^ PTSMT. Fluorodeoxyglucose (^18^F) positron emission tomography-CT demonstrated masses in both lungs, liver, and the colosigmoid junction. Colonoscopy showed multiple nodular mucosal tumors with erythematous margins and a maximum of 1.5 cm in diameter (**Figure 2A**) that were histologically confirmed as latency type III (34) EBV^+^ PTSMT. Immunohistology revealed low proliferating (Ki-67 <5%) spindle cells with expression of calponin and caldesmon as well as EBNA-2, lacking the expression of CD34 and S100. Furthermore, positivity for EBER was detected by *in situ* hybridization (**Figures 2B–F**). Moreover, PCR analysis revealed EBV viremia (EBV DNA copies: 43,258 U/ml). HIV serology was negative; lymphocyte counts and quantitative immunoglobin levels were in range throughout the medical history. No opportunistic or recurrent infections were reported prior to the heart transplantation, and therefore we clinically rule out HIV-SMT or CI-SMT as differential diagnoses.

Therapy initiation was complicated by severe sepsis, fungal pneumonia, and third-stage acute kidney injury [Kidney Disease: Improving Global Outcomes (KDIGO) guidelines (35)] with intermittent hemodialysis, drug-induced torsade de pointes, and resuscitation. Upon clinical stabilization, we initiated therapy with rituximab and reduced the doses of everolimus and tacrolimus, aiming for trough blood levels of 2–4 ng/ml for each drug. After four courses, thoracic CT revealed partial remission. EBV copies also decreased during therapy to undetectable numbers. Clinically, however, the patient suffered from severe pain of the upper right abdomen, and abdominal magnetic resonance imaging showed multiple new lesions of the liver and spleen.

Interferon-gamma enzyme-linked immuno spot (IFN-γ-EliSPOT) assay of the patient’s T cells revealed lack of reactivity against EBNA-1 in our patient, with 1.5 spots/250,000 peripheral blood mononuclear cells. This indicated an immunological gap against EBV and the PTSMT. Lacking other therapeutic options in the case, we decided to offer adoptive transfer of EBV-specific T lymphocytes to the patient as compassionate use. Informed consent was obtained from the patient using shared decision-making. The patient and her family explicitly consented to future publications regarding her case. The compassionate use of this novel therapeutic regimen was in accordance with the ethical standards of our institution and the Declaration of Helsinki. No other participants were included.

## Therapy and Results

We selected T cells exhibiting high reactivity against EBNA-1 and a pool of 43 major histocompatibility complex (MHC) class I and class II-restricted peptides from 15 different EBV proteins (PepTivator^®^ EBV EBNA-1 and PepTivator^®^ EBV Select, Miltenyi Biotec, Germany) from an unrelated donor from the alloCELL VST donor registry (36), exhibiting a 5/10 HLA allele match with the patient and a 1/10 HLA allele match with the transplanted heart to avoid cytotoxicity by posttransplant HLA antibodies (**Table 1**). The VSTs were manufactured using IFN-γ-based CliniMACS cytokine capture system as previously described (5).

**Table 1:** HLA genotypes of patient, heart allograft, and T-cell donor of ACT.

ID/HLA allele	A	A	B	B	C	C	DRB1	DRB1	DQB1	DQB1	Match
**Patient**	01	**03**	08	**35**	**04**	07	**01**	11:02	**05:01**	03:01	**5/10**
**Heart transplant**	02	24	52	**35**	12:02		09:01	11	03:03	03(7)	**1/10**
**VST cell donor**	03:01	**03:01**	35:01	**35:01**	**04:01**	04:01	**01:01**	01:01	**05:01**	05:01	

ACT, adoptive cell transfer; VST, virus-specific T cells; HLA, human leukocyte antigen; bold values indicate HLA allele matches.

Starting in September 2020, ACT was repeated every 2–4 weeks for a total of five infusions.

First, 1 × 10^4^ CD3^+^ T cells/kg body weight (BW) with a purity of 38.5% CD3^+^/IFN-γ^+^ EBV-specific T cells with a CD4/CD8 ratio of 3:1 were administered. Upon good tolerability, we increased dosing to 1.8 × 10^4^ CD3^+^ cells/kg BW.

Transfusion of EBV-specific VST was generally well-tolerated. Treatment-emergent adverse events (TEAEs) included third-grade anemia requiring transfusion of packed red blood cells (CTCAE v5.0), exacerbation of pain, nausea, and CRS upon increase in white blood cell counts. CRS presented with fever, hypotension responding to intravenous fluids, and hypoxia requiring low-flow oxygen *via* nasal cannula [ASBMT consensus grading 2 (37)]. Of those TEAEs, only CRS was attributed to the ACT; however, dose reductions regarding the ACT were not necessary. Of note, no signs of GvHD or transplant rejection were observed.

**Figure 3:**
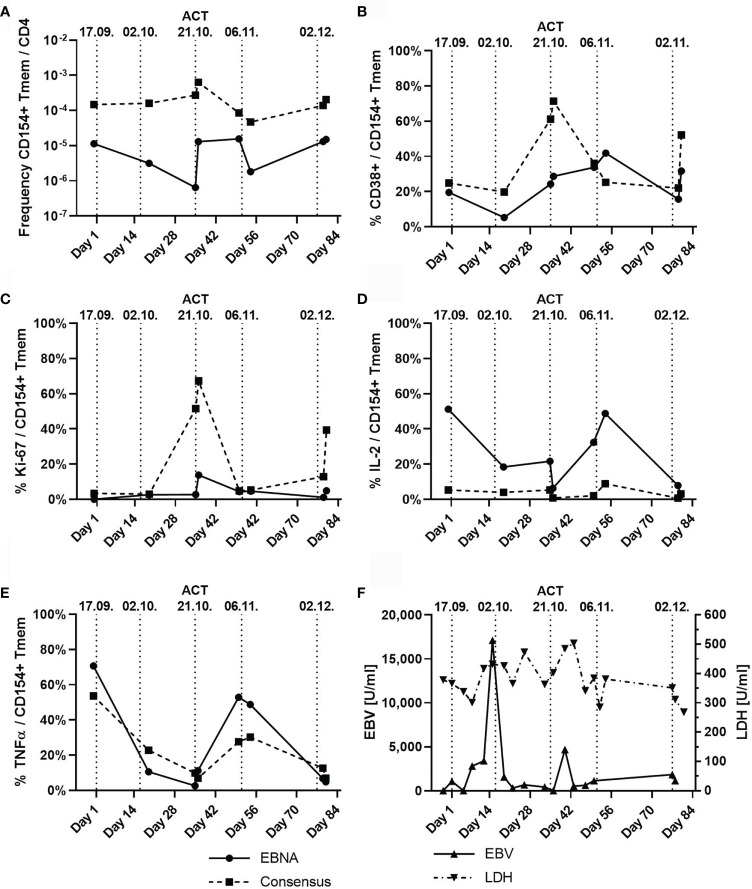
T-cell subpopulations and dynamic of Epstein–Barr virus (EBV) viremia and lactate dehydrogenase (LDH) during the course of five adoptive cell transfers (ACTs), indicated by vertical dotted lines. **(A)** Frequency of CD154^+^ (CD40L^+^) memory T cells (T_mem_) normalized to the count of CD4^+^ T cells with specificity to either EBNA (solid line) or EBV consensus antigens (dotted line). **(B)** Percentage of CD38^+^ T cells among CD154^+^ (CD40L^+^) T_mem_ cells depicted in panel **(A)**. **(C)** Percentage of Ki-67-expressing T cells among CD154^+^ (CD40L^+^) T_mem_ cells. **(D)** Percentage of Interleukin 2 (IL-2)-expressing T cells among CD154^+^ (CD40L^+^) T_mem_ cells. **(E)** Percentage of tumor necrosis factor alpha (TNF-α)-expressing T cells among CD154^+^ (CD40L^+^) T_mem_ cells. **(F)** Serum levels of EBV (U/ml) and LDH (U/ml). Remarks: Graphs in panels **(A–E)** are given for EBNA-1-specific T cells and T cells reactive against the EBV consensus pool of antigens.

To determine the immunological effects of ACT, blood samples taken before and after ACT were analyzed for EBV-reactive T-cell responses using antigen-reactive T-cell enrichment (ARTE), as previously described (38–42). Since the transferred EBV-specific T-cell product had a CD4/CD8 ratio of 3:1, we focused on specific CD4^+^ T cells. Frequencies of EBNA-1 and EBV consensus pool (PepTivator^®^ EBV EBNA-1 and PepTivator^®^ EBV Consensus, Miltenyi Biotec, Germany) reactive T cells were determined after 7 h of antigen stimulation and magnetic enrichment of CD40L^+^ (CD154^+^) CD4^+^ T cells (ARTE). Frequencies were calculated based on the relative cell count of CD40L^+^ (CD154^+^) memory T cells (T_mem_) after gating (**Supplementary Figure S1**) for CD4^+^ T cells. Quantification of the activation markers Ki-67 and CD38 was performed, and expression of the T-cell cytokines tumor necrosis factor (TNF)-α, IFN-γ, and interleukin (IL)-2 was measured to obtain a broad overview of T-cell activation and functionality. We found an increase in the frequency of CD40L^+^ (CD154^+^) T cells after the third ACT that was accompanied with increased expression of CD38 and Ki-67 and of TNF-α and IL-2 after the fourth ACT (**Figure 3**). Notably, *in vivo* expanded T cells displayed reactivity for both EBNA and EBV consensus pool antigens. An increase of IFN-γ^+^/CD40L^+^/CD4^+^ T cells was detected only with reactivity for EBNA antigens, while no increase of cells with specificity for EBV consensus pool was identified. Accordingly, we found increasing blood levels of IL-6 from initially 44 ng/l (day 6) up to 148 ng/l (day 37). We also detected suppression of EBV replication below the quantifiable detection threshold. Lactate dehydrogenase (LDH) levels decreased to physiological levels after the fifth ACT (**Figure 3**) (43–45). Additionally, we confirmed presentation and recognition of immunodominant EBNA-1 epitopes by the shared HLA alleles using the *in silico* prediction algorithms SYFPEITHI and NetMHC (**Supplementary Tables S2–S5**) (43–45).

Abdominal sonography revealed stable disease at 3 months of treatment initiation. At that point, the patient’s condition had improved so that we were able to discharge her with regular follow-up as an outpatient. However, 4 months after treatment initiation, the patient’s performance status dramatically decreased and no further treatment attempt was solicited by the patient. Unfortunately, the patient deceased due to progressing hepatic failure.

## Discussion

To our knowledge, we present the first report of ACT of EBV-specific T cells for a patient with PTSMT. ACT has previously been successfully implemented for patients with PTLD (1–5), CMV (4, 6), or HAdV infection (4, 7–9, 11) and suggested for PTSMT treatment (28).

Treatment with VST in this case of a young, heavily pretreated patient with an unfavorable prognosis of multilocular PTSMT resulted in stable disease at 3 months and overall survival of 4 months.

The patient succumbed to progressive liver failure most likely due to progressive disease of PTSMT. Importantly, during ACT, no safety signal regarding liver function tests was observed, and liver function tests deteriorated upon withdrawal of ACT. Liver failure due to EBV reactivation without tumor progression is unusual (46–48). However, association of hepatic failure to ACT or EBV reactivation cannot entirely be ruled out.

During the course of therapy, we detected an increase of EBNA-1 and EBV consensus pool reactive T cells with enhanced activity and a decrease in serum levels of LDH and EBV viremia. Increase of IL-6 levels was in line with activation of immune effector cells by EBV-specific donor T cells (49), which was clinically associated with a mild second-grade CRS (37).

In order to achieve maximum therapeutic efficacy for future patients, we would like to address some aspects that we consider pivotal when applying VST therapies.

In our case, five doses of each 1 × 10^4^–1.8 × 10^4^ CD3^+^ T cells/kg BW with a purity of 38.5% CD3^+^/IFN-γ^+^ EBV-specific T cells and a CD4/CD8 ratio of 3:1 were infused every 19 days on average (range: 16–27 days). With regard to previously published dosages of ACT (**Supplementary Table S1**), this shows that while lower cell counts for ACT may work, therapy response may correlate with the composition of T-cell subsets, such as ratio of CD4^+^ T cells (1).

Another critical issue is frequency of ACT of VST for antineoplastic therapy. Little is known regarding the optimal frequency, since comparative analyzes are lacking. We chose to quantify “pharmacokinetics” of ACT by assessment of immune effector cells, EBV viremia, and LDH. Nevertheless, it can be assumed that a higher frequency of ACT would be necessary to improve expansion and persistence of VST *in vivo*. On the other hand, limited availability of cell products confines therapy schedules. Therapy frequency should be adapted to tolerability, efficacy, and the individual VST cell counts in the patient, ideally accompanied by advanced cell monitoring, as suggested in this case report, to quantify T-cell activation and functionality.

To address the limited availability and dosing, tabelecleucel (tab-cel^®^, ATA129), an “off-the-shelf” allogenic T-cell immunotherapy, is investigated for EBV-positive leiomyosarcomas (NCT04554914) at doses of 2 × 10^6^ EBV-specific cytotoxic T cells/kg BW administered in 35 days lasting cycles on days 1, 8, and 15 for up to 24 months. The doses to be applied in the trial are notably higher than those in our case report, possibly leading to increased T-cell expansion and persistence. However, tabelecleucel contains *in vitro* expanded cell lines and it is currently unknown how numbers and function compare to directly *ex vivo* selected cells.

HLA matching is crucial in ACT of T cells to prevent GvHD, transplant rejection, and depletion of the transferred cells. Previous studies reported good results for PTLD and CMV infection treatment with 5/10 (5), 8/10 (3), and 9/10 (6) HLA matching. In a long-term follow-up report comprising of 33 cases of ACT-treated PTLD, a correlation of HLA matching and treatment response was found (1). In our case, the third-party T-cell donor matched 5/10 with the HLA genotype of the patient and 1/10 with the HLA genotype of the heart (**Table 1**). The selected donor offered a compromise between HLA compatibility with patient and solid organ transplant and frequency and distribution of EBV-specific T cells. The distinct mismatch between the HLA genotype of the heart and the donor was chosen to avoid elimination of transplanted T cells by HLA antibodies that were *bona fide* existent after heart transplantation and to also prevent *de novo* donor-specific antibody induction by the activated T cells.

The absence of severe adverse immune reactions or transplant rejection was possibly facilitated by continuing the administration of tacrolimus and everolimus, albeit in a reduced dose. Tacrolimus inhibits calcineurin, a protein phosphatase playing an important role in T-cell cytokine transcription and release. Everolimus, by contrast, inhibits the mTOR, a key kinase in cell cycle progression, leading to impaired T-cell activation and cell cycle arrest (50). Since both drugs decrease T-cell activity, the potential effect of the transfer of EBV-specific T cells can be diminished by high serum levels of these drugs. We attempted to mitigate this by dose adaptation for both drugs, but more efficacious effects may have been possible in the absence of tacrolimus and everolimus.

Previous work suggests another approach: sirolimus, the predecessor of everolimus, was able to induce and sustain complete remission in PTSMT patients (28, 30, 31). Interestingly, these cases have in common that the immunosuppressive therapy prior to the switch to sirolimus did not include rapalogs. Sirolimus and everolimus differ mostly in pharmacokinetic and not pharmacodynamic properties (51). Furthermore, everolimus inhibits SMC proliferation more efficaciously than sirolimus (52), which indicates that a treatment attempt with sirolimus will probably fail when PTSMT evolves in patients under rapalog treatment like ours.

Another approach in adoptive T-cell transfer is the combination with immune checkpoint inhibitors. Albeit the promising results from chimeric antigen receptor T cell (CAR-T cell) therapy (53), tolerability in transplanted patients is very low, with transplant rejection rates of 39.8% for solid transplants (54) and fatal GvHD in 7.7% for patients who underwent allogenic stem cell transplantation (55).

In this case report, we were able to show for the first time that ACT of EBV-specific T cells is a safe therapy option for PTSMT patients with an encouraging signal of effectiveness. We were able to dissect effectiveness by identification of T-cell proliferation, cytokine release, clearance of EBV from the peripheral blood and radiographic findings. Further studies are needed in order to explore this novel therapeutic option for PTSMT patients.

## Patient’s Perspective

Upon treatment initiation, and due to the lack of other suitable therapy options, the patient was in hopeful anticipation of a novel treatment approach, although she was clearly worried about TEAEs in light of her medical history. Using shared decision-making tools, we decided to move forward with the ACT and identified discharge from hospital with treatment as an outpatient as treatment objective.

We achieved that goal after three courses of ACT. Although we feared the long distance between the patient’s home and our treatment center would be a hindrance, we found her mental state and compliance much improved upon readmission to hospital. With the help of intensive physiotherapeutic therapy, we were able to discharge her again after five courses of ACT, where she continued to benefit from therapy for about a month. Unfortunately, 1 month after discharge, her physical and mental condition decreased dramatically and the patient expressed no wish in continuing medical therapy. Shortly after, the patient deceased due to hepatic failure. For this case report, we had the patient’s explicit consent to publish this report and thankfully received written informed consent from the patient’s father.

## Data Availability Statement

The datasets generated and analyzed for this case report can be acquired from the corresponding author.

## Ethics Statement

Informed consent was obtained from the patient using shared-decision-making. The patient and her family explicitly consented to future publications regarding her case. The compassionate use of this novel therapeutic regimen was in accordance with the ethical standards of our institution and the declaration of Helsinki. No other participants were included.

## Author Contributions

CB, LF, and B-TH designed the therapeutic setup. CB, LF, B-TH, and SH treated the patient. B-TH prepared the article. PB provided immunological data. WK and KK provided immunohistochemical analyses. BE-V and BM-K gave advice, provided the EBV-specific T cells, and performed *in silico* analyses. LF revised the article. All authors contributed to the article and approved the submitted version.

## Funding

B-TH was funded by the Deutsche Forschungsgemeinschaft (DFG, German Research Foundation) – project number 413490537 within the Kiel Clinician Scientist Programme in Evolutionary Medicine (CSEM).

## Conflict of Interest

The authors declare that the research was conducted in the absence of any commercial or financial relationships that could be construed as a potential conflict of interest.

## Publisher’s Note

All claims expressed in this article are solely those of the authors and do not necessarily represent those of their affiliated organizations, or those of the publisher, the editors and the reviewers. Any product that may be evaluated in this article, or claim that may be made by its manufacturer, is not guaranteed or endorsed by the publisher.
