# Supplementary material for: Adoptive Cell Transfer of Allogeneic Epstein–Barr Virus-Specific T Lymphocytes for Treatment of Refractory EBV-Associated Posttransplant Smooth Muscle Tumors: A Case Report
Source: Front Immunol. 2021 Dec 3;12:727814. doi: 10.3389/fimmu.2021.727814 (PMC8677671; doi:10.3389/fimmu.2021.727814)
Supplement: Supplementary file 1 [file DataSheet_1.docx]

Supplementary Material

# Supplementary Tables

**Supplementary Table 1:** Previously published ACT dosages.

| **Reference** | **Disease** | **Platform** | **Dosage** | **Frequency** |
| --- | --- | --- | --- | --- |
| Lindemann et al. (6) | CMV infection | CliniMACS (GMP PepTivator HCMVpp65) + IFN-γ  Cytokine Capture System | 2.5 × 10^4^ CMV-specific T cells / kg BW | Once |
| Mika et al. (3) | PTLD | CliniMACS (GMP PepTivator EBV-EBNA1 and EBV-Select) + IFN-γ Cytokine Capture System | 0.5 - 1 × 10^4^  EBV-specific T cells / kg BW | Every 1 1/3 months for a total of 6 ACT |
| Schultze-Florey et al. (5) | CNS PTLD | CliniMACS (ppEBNA1 and ppSelect)  + IFN-γ Cytokine Capture System | 2.5 × 10^4^  EBV-specific T cells / kg BW | Every 3 weeks for a total of 5 ACT |
| Schultze-Florey et al. (7) | HAdV infection | CliniMACS (GMP PepTivator AdV5 Hecon and AdV5 Penton) + IFN-γ Cytokine Capture System | 1.6 × 10^3^ - 1 × 10^4^  HAdV-specific T cells / kg BW | Every 3-4 weeks  for a total of 10 ACT |

**Supplementary Table 2:** In silico prediction of immunodominant EBNA-1 epitopes presented by the shared HLA alleles HLA-A*03:01. Regarding prediction analysis by SYFPEITHI, the top 2% from the prediction list are shown. Rank thresholds for NetMHC analysis was 0.5 for strong and 2.0 for weak binding peptides.

| **HLA-A*03:01** | | | |
| --- | --- | --- | --- |
| **Position** | **Sequence** | **Method** | **SYNFPEITHI Score / NetMHC Binding Strength** |
| **Nonamers** | | | |
| 578 | A I K D L V M T K | SYFPEITHI | 28 |
| 506 | G V F V Y G G S K | SYFPEITHI | 26 |
| 553 | P L R E S I V C Y | SYFPEITHI | 24 |
| 487 | A L L A R S H V E | SYFPEITHI | 23 |
| 488 | L L A R S H V E R | SYFPEITHI | 22 |
| 508 | F V Y G G S K T S | SYFPEITHI | 22 |
| 358 | R E R A R G G S R | SYFPEITHI | 21 |
| 519 | N L R R G T A L A | SYFPEITHI | 21 |
| 67 | R D G V R R P Q K | SYFPEITHI | 20 |
| 13 | G L G E K G D T S | SYFPEITHI | 19 |
| 45 | R G R G R G G G R | SYFPEITHI | 19 |
| 459 | R K K G G W F G K | SYFPEITHI | 19 |
| 505 | G V F V Y G G S K | NetMHC | 0.4 |
| 577 | A I K D L V M T K | NetMHC | 0.4 |
| 513 | K T S L Y N L R R | NetMHC | 1.1 |
| **Decamers** | | | |
| 487 | A L L A R S H V E R | SYFPEITHI | 25 |
| 532 | R L T P L S R L P F | SYFPEITHI | 25 |
| 484 | G L R A L L A R S H | SYFPEITHI | 24 |
| 519 | N L R R G T A L A I | SYFPEITHI | 23 |
| 516 | S L Y N L R R G T A | SYFPEITHI | 22 |
| 370 | R G R G R G R G E K | SYFPEITHI | 21 |
| 74 | Q K R P S C I G C K | SYFPEITHI | 19 |
| 451 | R G Q G D G G R R K | SYFPEITHI | 19 |
| 505 | A G V F V Y G G S K | SYFPEITHI | 19 |
| 508 | F V Y G G S K T S L | SYFPEITHI | 19 |
| 553 | P L R E S I V C Y F | SYFPEITHI | 18 |
| 577 | D A I K D L V M T K | SYFPEITHI | 18 |
| 504 | A G V F V Y G G S K | NetMHC | 0.4 |
| 566 | Q T H I F A E V L K | NetMHC | 1.0 |
| 486 | A L L A R S H V E R | NetMHC | 1.2 |

**Supplementary Table 3:** In silico prediction of immunodominant EBNA-1 epitopes presented by the shared HLA alleles HLA-B*35:01. Regarding prediction analysis by SYFPEITHI, the top 2% from the prediction list are shown. Rank thresholds for NetMHC analysis was 0.5 for strong and 2.0 for weak binding peptides.

| **HLA-B*35:01** | | | |
| --- | --- | --- | --- |
| **Position** | **Sequence** | **Method** | **SYNFPEITHI Score / NetMHC Binding Strength** |
| **Nonamers** | | | |
| 407 | H P V G E A D Y F | SYFPEITHI | 21 |
| 528 | I P Q C R L T P L | SYFPEITHI | 21 |
| 546 | G P G P Q P G P L | SYFPEITHI | 20 |
| 397 | R P P P G R R P F | SYFPEITHI | 18 |
| 398 | P P P G R R P F F | SYFPEITHI | 18 |
| 426 | E P D V P P G A I | SYFPEITHI | 18 |
| 72 | R P Q K R P S C I | SYFPEITHI | 17 |
| 550 | Q P G P L R E S I | SYFPEITHI | 17 |
| 477 | K F E N I A E G L | SYFPEITHI | 14 |
| 562 | M V F L Q T H I F | NetMHC | 0.1 |
| 406 | H P V G E A D Y F | NetMHC | 0.15 |
| 501 | T W V A G V F V Y | NetMHC | 0.4 |
| 609 | F P P M V E G A A | NetMHC | 0.7 |
| 408 | V G E A D Y F E Y | NetMHC | 1.1 |
| 527 | I P Q C R L T P L | NetMHC | 1.2 |
| 538 | L P F G M A P G P | NetMHC | 1.3 |
| 509 | Y G G S K T S L Y | NetMHC | 1.5 |
| 507 | F V Y G G S K T S | NetMHC | 1.8 |
| 556 | S I V C Y F M V F | NetMHC | 1.9 |
| 382 | S P S S Q S S S S | NetMHC | 2.0 |
| **Decamers** | | | |
| 552 | G P L R E S I V C Y | SYFPEITHI | 24 |
| 5 | G P G T G P G N G L | SYFPEITHI | 20 |
| 397 | R P P P G R R P F F | SYFPEITHI | 18 |
| 534 | T P L S R L P F G M | SYFPEITHI | 17 |
| 407 | H P V G E A D Y F E | SYFPEITHI | 13 |
| 422 | G P D G E P D V P P | SYFPEITHI | 13 |
| 479 | E N I A E G L R A L | SYFPEITHI | 13 |
| 566 | L Q T H I F A E V L | SYFPEITHI | 13 |
| 552 | G P L R E S I V C Y | SYFPEITHI | 24 |
| 405 | F H P V G E A D Y F | NetMHC | 0.6 |
| 609 | F P P M V E G A A A | NetMHC | 1.3 |
| 561 | F M V F L Q T H I F | NetMHC | 1.5 |
| 538 | L P F G M A P G P G | NetMHC | 1.5 |
| 404 | F F H P V G E A D Y | NetMHC | 1.6 |
| 533 | T P L S R L P F G M | NetMHC | 1.6 |

**Supplementary Table 4:** In silico prediction of immunodominant EBNA-1 epitopes presented by the shared HLA alleles HLA-C*04:01. Rank thresholds for NetMHC analysis was 0.5 for strong and 2.0 for weak binding peptides.

| **HLA-C*04:01** | | | |
| --- | --- | --- | --- |
| **Position** | **Sequence** | **Method** | **NetMHC Binding Strength** |
| **Nonamers** | | | |
| 357 | R E R A R G G S R | NetMHC | 0.25 |
| 477 | F E N I A E G L R | NetMHC | 0.4 |
| 396 | R P P P G R R P F | NetMHC | 0.6 |
| 562 | M V F L Q T H I F | NetMHC | 0.9 |
| 365 | R E R A R G R G R | NetMHC | 1.0 |
| 605 | L P P W F P P M V | NetMHC | 1.1 |
| 604 | D L P P W F P P M | NetMHC | 1.1 |
| 517 | Y N L R R G T A L | NetMHC | 1.1 |
| 545 | G P G P Q P G P L | NetMHC | 1.2 |
| 400 | G R R P F F H P V | NetMHC | 1.5 |
| 608 | W F P P M V E G A | NetMHC | 1.9 |
| 500 | G T W V A G V F V | NetMHC | 2.0 |

**Supplementary Table 5:** In silico prediction of immunodominant EBNA-1 epitopes presented by the shared HLA alleles DRB1*01:01. The top 2% from the prediction list are shown.

| **HLA-DRB1*01:01** | | | |
| --- | --- | --- | --- |
| **Position** | **Sequence** | **Method** | **SYNFPEITHI Score** |
| **15-mers** | | | |
| 560 | C Y F M V F L Q T H I F A E V | SYFPEITHI | 32 |
| 515 | T S L Y N L R R G T A L A I P | SYFPEITHI | 29 |
| 538 | R L P F G M A P G P G P Q P G | SYFPEITHI | 29 |
| 482 | A E G L R A L L A R S H V E R | SYFPEITHI | 27 |
| 505 | A G V F V Y G G S K T S L Y N | SYFPEITHI | 27 |
| 536 | L S R L P F G M A P G P G P Q | SYFPEITHI | 27 |
| 576 | K D A I K D L V M T K P A P T | SYFPEITHI | 27 |
| 517 | L Y N L R R G T A L A I P Q C | SYFPEITHI | 26 |
| 465 | F G K H R G Q G G S N P K F E | SYFPEITHI | 25 |
| 530 | Q C R L T P L S R L P F G M A | SYFPEITHI | 25 |
| 431 | P G A I E Q G P A D D P G E G | SYFPEITHI | 24 |
| 479 | E N I A E G L R A L L A R S H | SYFPEITHI | 24 |

# Supplementary Figures


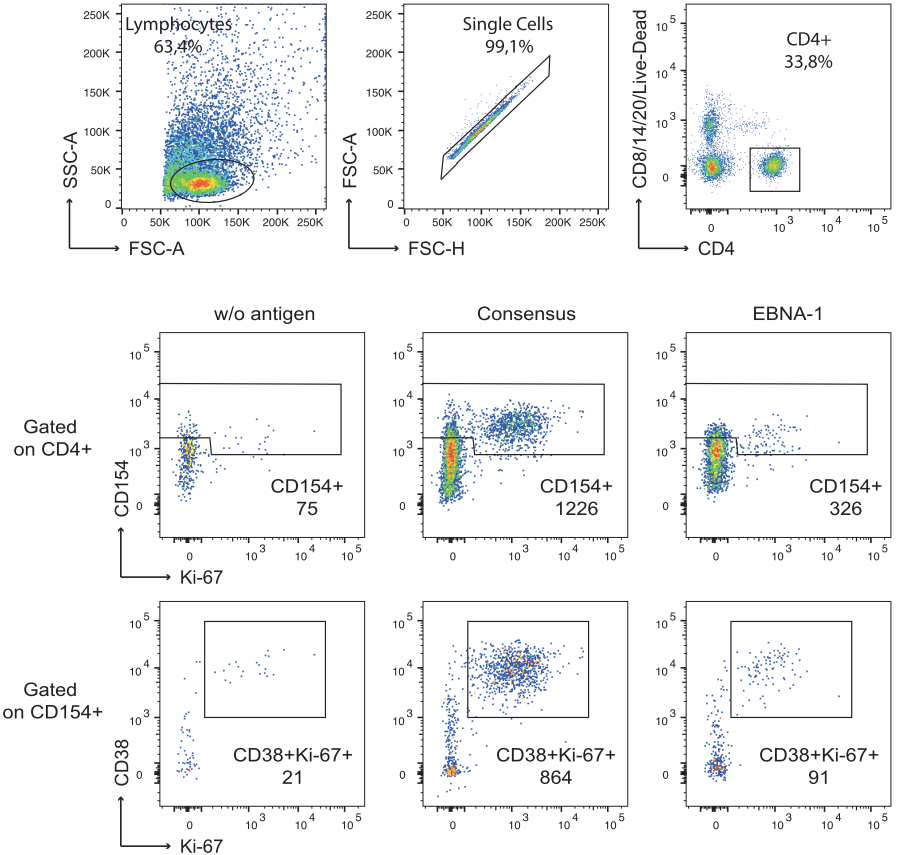


Supplementary Figure 1: Gating strategy for detection of EBV-reactive T cells following magnetic enrichment of CD154^+^ (CD40L^+^) T cells from 1 × 10^7^ input PBMCs. Cells were gated on lymphocytes, single cells and CD8, CD14, CD20 and dead cells were excluded to identify CD4^+^ T cells. Antigen-specific cells are defined as CD154^+^ and the co-staining with the recent proliferation makers Ki-67 and CD38 is shown. Numbers indicate absolute cell numbers of CD154^+^ (CD40L^+^) or Ki-67^+^ CD38^+^ cells following magnetic enrichment.
